# The Effect of Perceived Stress and Digital Literacy on Student Satisfaction with Distance Education

**DOI:** 10.1590/1980-220X-REEUSP-2021-0488en

**Published:** 2022-09-02

**Authors:** Sevgi Vermisli, Esra Cevik, Celalettin Cevik

**Affiliations:** 1 Bursa City Hospital, Operating Room, Bursa, Turkey.; 2 Balikesir University, Faculty of Health Sciences, Department of Midwifery, Balikesir, Turkey.; 3 Balikesir University, Faculty of Health Sciences, Department of Public Health Nursing, Balikesir, Turkey.

**Keywords:** Computer Literacy, Distance Education, Stress, Physiological, Covid-19, Students, Personal Satisfaction, Alfabetização Digital, Educação à Distância, Estresse Fisiológico, Covid-19, Estudantes, Satisfação Pessoal, Alfabetización Digital, Educación a Distancia, Estrés Fisiológico, Covid-19, Estudiantes, Satisfacción Personal

## Abstract

**Objective:**

To examine the relationship between perceived stress/digital literacy and student satisfaction in health science college students in the distance education process.

**Method:**

The cross-sectional study was conducted by collecting data from 842 students. The dependent variable was student satisfaction in distance education. For the analyses, t-test, ANOVA (post hoc: Bonferroni), and linear regression methods were used.

**Results:**

Distance education student satisfaction was 178.21 ± 48.64. Student satisfaction was low among those who think that distance education is not more effective than face-to-face education, live in villages/towns, and have high perceived stress. Student satisfaction was high among those who do not have limited internet access, can access the internet via computer, follow the lessons regularly every week, think distance education is applicable in the health domain, can acquire instant feedback from the instructor, and have increased digital literacy.

**Conclusion:**

Distance education student satisfaction was found to be moderate. Student satisfaction in distance education increases as perceived stress levels decrease and digital literacy levels increase.

## RESUMO

**Objetivo:**

Examinar a relação entre estresse percebido e letramento digital com a satisfação de alunos de faculdades de ciências da saúde no processo de educação a distância.

**Método:**

O estudo transversal foi realizado por meio da coleta de dados de 842 alunos. A variável dependente foi a satisfação dos alunos na educação a distância. Para as análises, foram utilizados os métodos de teste t, ANOVA (post hoc: Bonferroni) e regressão linear.

**Resultados:**

A satisfação dos alunos de educação a distância foi de 178,21 ± 48,64. A satisfação dos alunos foi baixa entre aqueles que pensam que a educação a distância não é mais eficaz do que a educação presencial, que moram em vilas/cidades e apresentam alto estresse percebido. A satisfação dos alunos foi alta entre aqueles que não têm acesso limitado à internet, podem acessar a internet pelo computador, acompanhar as aulas regularmente toda semana, acham que a educação a distância é aplicável no domínio da saúde, podem adquirir feedback instantâneo do instrutor e têm maior letramento digital.

**Conclusão:**

A satisfação dos alunos de educação a distância foi considerada moderada. A satisfação dos alunos na educação a distância aumenta à medida que os níveis de estresse percebido diminuem e os níveis de letramento digital aumentam.

## RESUMEN

**Objetivo:**

Examinar la relación entre el estrés percibido y la alfabetización digital con la satisfacción de los estudiantes de la facultad de ciencias de la salud en el proceso de educación a distancia.

**Método:**

El estudio transversal se realizó mediante la recopilación de datos de 842 estudiantes. La variable dependiente fue la satisfacción de los estudiantes en educación a distancia. Para los análisis, se utilizaron métodos de prueba t, ANOVA (post hoc: Bonferroni) y regresión lineal.

**Resultados:**

La satisfacción de los estudiantes de educación a distancia fue de 178,21 ± 48,64. La satisfacción de los estudiantes fue baja entre aquellos que piensan que la educación a distancia no es más efectiva que la educación presencial, los que viven en aldeas/pueblos y con un alto nivel de estrés percibido. La satisfacción de los estudiantes fue alta entre aquellos que no tienen acceso limitado a Internet, pueden acceder a Internet a través de una computadora, siguen las lecciones con regularidad todas las semanas, piensan que la educación a distancia es aplicable en el ámbito de la salud, pueden obtener retroalimentación instantánea del instructor y tienen un mayor conocimiento de alfabetización digital.

**Conclusión:**

Se encontró que la satisfacción de los estudiantes de educación a distancia es moderada. La satisfacción de los estudiantes en la educación a distancia aumenta a medida que disminuyen los niveles de estrés percibido y aumentan los niveles de alfabetización digital.

## INTRODUCTION

One of the areas most affected by the Covid-19 pandemic is the education and training systems. All over the world, face-to- face education has been discontinued and transition to distance education (DE) at all levels of education was implemented^(1)^. During the DE process, students stayed away from their friends and educational environments. As a result, social and economic life became constrained^(2)^.

Digital literacy is the ability to find, understand, analyze, produce, and share information through smartphones, tablets, laptops, and desktop computers^(3)^. Students must have high levels of digital literacy to conduct procedures efficiently^(4)^.

Student satisfaction (SS) is the satisfaction in learning activities and services among students; it plays a major role in shaping the quality of education and preserving the aspect of learning^(5)^. Determining learning satisfaction and factors affecting health science students who continue their education with DE programs during the pandemic is important in terms of improving program outcomes and educational quality^(6)^.

In the literature, there are studies demonstrating that DE satisfaction of university students during the pandemic is at low^(1,7)^, medium^(8)^, and high^(9,10)^ levels. In DE process, SS appears to be affected by age, marital status^(11)^, gender^(8,11,12)^, learning conditions^(12)^, university entrance field^(10,11)^, computer skills^(13)^, problems related to systems^(14,15)^, and internet access via Wi-Fi^(8)^. Student satisfaction plays an important role in the continuation of learning^(13)^. The perceived stress during the pandemic process and the digital literacy skills that students need more intensely than before the pandemic are important for the effective continuation of learning. In this transition period, a comprehensive evaluation could not be made to evaluate students’ adaptation to distance education and the effectiveness of distance education^(10)^. When the literature was reviewed, there were no studies that investigated the association between perceived stress/digital literacy levels and SS in distant education. The object of the research is to examine the relationship between perceived stress/digital literacy and SS in health science faculty students in the DE process.

## METHOD

### Design of Study

The cross-sectional study was conducted between March and May 2021 in Turkey. The study’s population included 1,056 students from the departments of nursing, midwifery, and physical therapy and rehabilitation at Balıkesir University Faculty of Health Sciences (BUFHS).

### Population

In a process where education was carried out remotely, it was not possible to meet students face-to-face. The sample size was not calculated in this study; all students who volunteered to participate and met the research entry criteria were contacted. The research sample consists of students from the BUFHS who have access to the online questionnaire, volunteer to participate in the study, and pursue their undergraduate education remotely during the Covid-19 pandemic. The link to the questionnaire form prepared online was delivered to the students via e-mail and social platforms.

The study sample consisted of 842 participants (79.7%) who completed all of the online forms. The study dependent variable was the level of satisfaction among distant education students using information technology. Sociodemographic characteristics, DE features, digital literacy, and perceived stress levels were the independent variables.

### Data Collection

#### Sociodemographic Characteristics Information Form

The form was developed by (our) researchers after a literature^(1,10,11)^ review. In the first part of the form, which consists of two parts, there were 10 questions about sociodemographic characteristics and 13 questions about DE.

#### Perceived Stress Scale (PSS)

An adaptation of a standardized scale^(16)^ was used to measure what situations in an individual’s life are perceived as stress. The PSS was adapted into Turkish by Eskin et al. in 2013 through a study with university students. In the validity study of the 10-item scale, the Cronbach’s 〈 coefficient was calculated as 0.82 for the overall PSS^(17)^. In this study, the Cronbach’s 〈 coefficient was calculated as 0.79 for the overall PSS. The scale has a total of 14 items and one dimension. Each item is rated on a five point Likert scale, between “0 (never)” and “4 (very often)”. The total score obtained from the scale ranges from 0 to 40, and an increase in the score indicates an increase in stress^(17)^.

#### Digital Literacy Scale (DLS)

An adaptation of a standardized scale was used^(18)^. The Digital Literacy Scale was adapted into Turkish by Hamutoğlu et al. in 2017 through a study with university students. In the scale validity study, the Cronbach’s 〈 coefficient was 0.93 for the “whole scale” subscale, 0.88 for the “attitude” subscale, 0.89 for the “technical” subscale, 0.70 for the “cognitive”, and 0.72 for the “social”^(4)^. In this study, the Cronbach’s 〈 coefficient was 0.96 for the “whole scale” subscale, 0.89 for the “attitude” subscale, 0.92 for the “technical” subscale, 0.72 for the “cognitive”, and 0.70 for the “social”^(4)^. The scale consists of 17 items and four dimensions^(4)^. It is evaluated on a five-point Likert scale ranging from “I strongly agree”^(5)^ to “I strongly disagree”^(1)^. The four-factor structure obtained from the scale can be evaluated as four different dimensions, and the total score related to digital literacy can be deduced. The total score obtained from the scale varies between 17 and 85, and an increase in the score indicates an increase in digital literacy^(4,18)^.

#### Information Technologies-Based Distance Education Students Satisfaction Scale

The original form of scale is in Turkish. The scale was developed by Kukul in 2011 through a study with university students. In the scale validity study, the Cronbach’s 〈 coefficient was 0.70 for the “whole scale” subscale, 0.93 for the “structure and operation of the program” subscale, 0.96 for the “Interaction” subscale, and 0.86 for the “Common problem areas” subscale^(4)^. In this study, the Cronbach’s 〈 coefficient was 0.98 for the “whole scale” subscale, 0.80 for the “attitude” subscale, 0.94 for the “the technical” subscale, 0.90 for the “cognitive”, and 0.88 for the “social”^(4)^. The scale consists of three factors and 42 items. It is evaluated on a five-point Likert scale between “strongly disagree”^(1)^ and “strongly agree”^(5)^. As the scale score increases, the level of satisfaction also increases. In total, the range of 42–92 points is the indicator of “very low” satisfaction; 93–143 points, of “low” satisfaction; 144–194 points, of “moderate” satisfaction; 195–245 points, of “high” satisfaction; and 246–294 points, of “very high” satisfaction^(13)^.

### Data Analysis

During data analysis, numbers, percentages, arithmetic mean, and standard deviation were used to compare the mean scores in independent groups; Student’s t-test and Bonferroni-corrected one-way variance analysis were also used. Multivariate linear regression analysis was used to investigate the relationship between the independent and dependent variables. All statistical analyses were performed using SPSS software version 26.

### Ethical Aspects

Permission was obtained from the Health Sciences School Dean’s Office (dated 07d.01m.2021y and numbered E.1027) and Clinical Research Ethics Committee (dated 04d.03m.2021y and numbered E.15.726, in Balıkesir University) before the study was conducted. The study have been performed in accordance with the ethical standards laid down in the Declaration of Helsinki (as revised in Brazil 2013). Informed consent was taken from all participants before starting the study and anonymity is preserved. Voluntary consent information about the study is included in the invitation to participate in the study, which is sent to the participants online. In line with this information, participants who agree to participate in the study are directed to the research page when they select the “I accept” option.

## RESULTS

The research group consisted of 842 participants. It was observed that 84.0% were women, 62.6% were nursing students, 62.1% were people whose income was equal to their expenses, and 38.8% were people living in the city center. Among the participants, 42.6% of them stated that they have a computer, 64.4% can easily enter the DE platform, 67.1% follow the lessons regularly every week, 68.3% state that DE is unapplicable in the health education, and 77.4% reported that DE will negatively affect their post-graduation practice skills (Table 1).

**Table 1.** Sociodemographic and DE characteristics of the research group (n = 842) – Balikesir Turkey, 2021.

Variables	n	%	Variables	n	%
**Age**			**Having a computer**		
18−20 years	446	53.0	No	484	57.5
21 +	396	47.0	Yes	358	42.5
**Sex**			**Limited internet access**		
Female	707	84.0	No	469	55.7
Male	135	16.0	Yes	373	44.3
**Department**			**Income**		
Midwifery	258	30.6	Income>expenses	110	13.1
Nursing	527	62.6	Income=expenses	523	62.1
PTR	57	6.8	Income<expenses	209	24.8
**Grade**			**Living place**		
1	202	24.0	City	327	38.8
2	211	25.1	District	284	33.7
3	215	25.5	Town	50	5.9
4	214	25.4	Village	181	21.5
**DE access tool**			**Regular follow-up of lessons**		
Telephone	423	50.2	Yes	565	67.1
Tablet	61	7.2	Partially	210	24.9
Computer	358	42.5	No	67	8.0
**Feedback from teachers**			**Pandemic is limiting access to the university library**		
Yes	324	38.5	Yes	239	28.4
Partially	361	42.9	Partially	236	28.0
No	157	18.6	No	367	43.6
**DE negatively affects practice skills**			**DE is applicable in the health education**		
Yes	652	77.4	Yes	77	9.1
Partially	108	12.8	Partially	190	22.6
No	82	9.7	No	575	68.3
**Covid-19 test**			**Family history of Covid-19**		
No	671	79.7	No	745	88.5
Yes	171	20.3	Yes	97	11.5
**Doing homework/ presentations in digital platform is difficult**			**DE is more effective than face-to-face education**		
Yes	340	40.4	Yes	72	8.6
Partially	271	32.2	Partially	115	13.7
No	231	27.4	No	655	77.8
**Theoretical lessons should continue with DE after the pandemic**			**Applied lessons should continue with DE after the pandemic**		
Yes	372	44.2	Yes	49	5.8
No	470	55.8	No	793	94.2

DE = Distance education, PTR = Physical therapy and rehabilitation.

The descriptive features of some continuous variables of the participants are given in Table 2. The average daily computer usage time was 3.56 ± 2.66/hour, average daily online time was 6.33 ± 4.30/hour, mean digital literacy score was 54.45 ± 14.49, mean score of perceived stress was 21.47 ± 5.78, mean score of DE SS was 178.21 ± 48.64 (Table 2).

**Table 2.** Descriptive features of some continuous variables in the research group (n = 842) – Balikesir, Turkey, 2021.

Variables	Mean ± SD	(Min–Max)
**Age**	20.71 ± 2.31	18−47
**Daily computer usage time/hours**	3.56 ± 2.66	1–15
**Lifetime computer usage /years**	5.70 ± 4.34	1−16
**Daily online time/hours**	6.33 ± 4.30	1−24
**Digital literacy**	54.45 ± 14.49	17−84
Attitude sub-dimension	21.82 ± 6.44	7−35
Technical sub-dimension	20.04 ± 5.69	6−30
Cognitive sub-dimension	6.55 ± 1.94	2−10
Social sub-dimension	6.01 ± 1.92	2–9
**Perceived stress**	21.47 ± 5.78	4-40
**DE SS**	178.21 ± 48.64	48–288
Structure and operation of the program sub-dimension	67.73 ± 21.94	16−112
Interaction sub-dimension	83.42 ± 28.57	20−140
Common problem areas sub-dimension	27.04 ± 9.72	6–42

SD = Standard deviation, DE = Distance education, SS = Student satisfaction.

The participants’ DE satisfaction scores according to the characteristics of sociodemographic and DE are given in Table 3. The satisfaction score was significantly higher for the midwifery, physiotherapy, and rehabilitation students (p = 0.002); the first and fourth-grade students (p = 0.000); those whose income is more than/equal to their expenses (p = 0.000); those living in city/town (p = 0.000); those with a computer (p = 0.000); those with no limited internet access (uninterrupted internet access/stable/good internet) (p = 0.000); those who have not undergone a Covid-19 test (p = 0.011); and those who do not have a family history of Covid-19 (p = 0.023) (Table 3).

**Table 3.** DE satisfaction scores of the research group according to the sociodemographic and DE characteristics (n = 842) – Balikesir, Turkey, 2021.

Variables	n	Mean ± SD	Test statistics	p
**Age**				
18–20 years	446	180.60 ± 44.70	t = 1.520	0.133
21 +	396	175.50 ± 52.65		
**Sex**				
Female	707	178.61 ± 46.64	t = 0.473	0.637
Male	135	176.10 ± 58.15		
**Department**				
Midwifery^a^	258	183.04 ± 45.22	F = 6.178	0.002
Nursing^b^	527	174.14 ± 50.59		a = c > b
Physiotherapy and rehabilitation^c^	57	193.94 ± 39.84		
**Grade**				
1^a^	202	190.75 ± 39.69	F = 10.148	0.000
2^b^	211	172.77 ± 46.39		a = d > b > c
3^c^	215	167.02 ± 48.82		
4^d^	214	182.96 ± 54.86		
**Income**				
Income > expense^a^	110	177.36 ± 39.49	F = 9.283	0.000
Income = expenses^b^	523	183.18 ± 51.03		a = b > c
Income < expenses^c^	209	166.21 ± 44.77		
**Living place**				
City^a^	327	189.70 ± 26.76	F = 30.731	0.000
Town^b^	284	185.03 ± 27.00		a = b > c = d
Town^c^	50	151.34 ± 10.06		
Village^d^	181	154.16 ± 17.90		
**Having a computer**				
No	347	168.00 ± 50.96	t = −5.176	0.000
Yes	495	185.36 ± 45.64		
**Limited internet access**				
No	469	188.02 ± 45.67	t = 6.737	0.000
Yes	373	165.86 ± 49.49		
**Covid-19 test**				
No	671	180.38 ± 48.35	t = 2.565	0.011
Yes	171	169.66 ± 48.94		
**Family history of Covid-19**				
No	745	179.47 ± 47.78	t = 2.281	0.023
Yes	97	167.63 ± 53.85		

SD = Standard deviation.

According to the results of Pearson correlation analysis, the satisfaction score of students in DE, based on information technologies, increases as lifetime computer usage time increases, as the level of digital literacy increases, and as the perceived stress level decreases (p = 0.000) (Table 4).

**Table 4.** Pearson correlation analysis of SS score in DE according to some variables (n = 842) – Balikesir, Turkey, 2021.

Values	r	p
**Age**	0.021	0.535
**Daily computer usage time**	0.029	0.404
**Lifetime computer usage time**	0.158	0.000
**Daily online time**	0.021	0.539
**Digital literacy total**	0.590	0.000
Attitude sub-dimension	0.571	0.000
Technic sub-dimension	0.537	0.000
Cognitive sub-dimension	0.488	0.000
Social sub-dimension	0.447	0.000
**Perceived stress**	−0.308	0.000

The evaluation of SS score in DE by linear regression analysis using backward elimination method is given in Table 5. The satisfaction score was low among those living in villages/towns, those who think DE is not more effective than face-to-face education, and those with high perceived stress (p < 0.000). The SS was high for those who do not have limited internet access (p = 0.009); those who can access the internet via computer (p = 0.026); those who regularly follow the lessons every week (p = 0.000); those who think that DE is applicable in the health education (p = 0.013); those who can acquire instant feedback from the instructor (p < 0.000); those who think that theoretical lessons should be conducted with DE after the pandemic (p = 0.002); and those with high digital literacy (p = 0.000) (Table 5).

**Table 5.** Evaluation of SS score in DE by linear regression analysis using backward elimination method (n = 842) – Balikesir, Turkey, 2021.

Variables	B	Std Beta	t	p	95% CI	
					Lower	Upper
**Living place**	−7.108	−0.167	−6.449	**0.000**	−9.27	−4.94
**Internet access limitation**	6.926	0.071	2.626	**0.009**	1.74	12.10
**DE access tool**	2.976	0.059	2.231	**0.026**	0.35	5.59
**Feedback from teachers**	18.515	0.278	10.475	**0.000**	15.04	21.98
**Digital literacy**	1.174	0.350	12.118	**0.000**	1.04	1.36
**Perceived stress**	−0.953	−0.113	−4.312	**0.000**	−1.38	–0.51
**Follow-up lesson regularly every week**	11.101	0.145	5.182	**0.000**	6.86	15.30
**DE is applicable in the health education**	5.558	0.074	2.495	**0.013**	1.18	9.92
**DE is more effective than face-to-face education**	−7.005	−0.089	−3.013	**0.003**	−11.56	−2.44
**Theoretical lessons should continue with DE after the pandemic**	8.662	0.088	3.167	**0.002**	3.29	14.03

F = 98.825, p = 0.000, Durbin Watson = 1.456, R^2^= 0.54 Adjusted R^2^= 0.52. CI = Confidence interval, DE = Distance education.

## DISCUSSION

Evaluation of SS and influencing factors are important for a qualified DE process. This research is one of the first studies to evaluate sociodemographic variables in health science college students, as well as the relationship between perceived stress/digital literacy levels and SS. In our research, students’ DE satisfaction was moderate. The literature showed that SS was low^(1,7,19)^, middle^(8)^, and high^(9,10)^.

In our research, the satisfaction of students living in the village/town was negatively affected, similar to the literature^(14,15)^. According to our results, parallel to the literature, it can be determined that inequalities regarding having important components of DE, such as computers, mobile phones, and the internet, are obstacles to achieving satisfaction in DE for students in rural areas. Eliminating inequalities in technological opportunities will play a key role in increasing SS.

Our study further determined that students’ thinking that DE is not more effective than face-to-face education negatively affects satisfaction. The literature reported that 49.7% of the students definitely did not prefer distance learning compared to face-to-face learning, and 43.8% were definitely not satisfied with DE^(5)^. Our findings are similar to the literature. Other studies during the pandemic have reported that theoretical lessons can be conducted with DE; however, there are problems with applied lessons^(14,15)^. Alternatively, it is stated that although DE has limitations for applied lessons, it can be effectively maintained, due to the creation of different strategies^(6)^. In the DE model, we believe that interaction problems encountered by students should be evaluated and educational environments should be organized to meet their learning needs, in addition to support students in this regard.

In our research, it was determined that the students’ perceived stress level score was at a moderate level and had a negative impact on satisfaction. Similar to our research, it was stated in the literature that the pandemic negatively affected the stress levels of university students^(20,21)^. It was reported that the DE system causes students to experience more stress^(22)^. It is also stated that exposure of students to long-term and uncontrollable stressors during the learning phase negatively affects both their professional identity development and health^(23)^. Reducing the stress levels of the health science college students, who will replace health workers at the forefront in the fight against the pandemic, is important in terms of both increasing their educational satisfaction, structuring their future in confidence, and fulfilling their duties and responsibilities professionally when they graduate.

According to our research, educational satisfaction of students who have unlimited internet access (uninterrupted/high- speed internet access) and can easily access the internet via computer is positively affected. The transition to DE was made without a comprehensive assessment of students’ DE opportunities at the beginning of the pandemic^(11)^. Disruptions due to insufficient infrastructure or internet access and hardware (computer, tablet, etc.) deficiencies are among the disadvantages of DE^(19)^. In the literature, it can be deduced that more than 60% of students are not ready for online classes due to poor network infrastructure and limited daily network data^(24)^. Another study reported that 65.8% of students experienced issues with their internet connection and the extra financial burden for internet quota caused further problems in DE^(25)^. A study conducted by Buluk and Esitti (2020) determined that 21.96% of students had problems in following classes due to lack of computers and/or other equipment, and 27.57% faced problems due to frequent disconnections^(12)^. The preparations proposed by UNESCO (2020) for DE during the Covid-19 pandemic are pedagogical preparation, content preparation, technological preparation, and monitoring and evaluation preparation^(26)^. The review of the adequacy of the internet and computer supports provided may be recommended to make necessary enhancements.

In our research, we determined that the satisfaction of students who follow regular lessons every week and believe that they can acquire feedback from the instructor at any moment is positively affected. In the literature, it was reported that, during the pandemic, 33.64% of students were able to follow the lessons completely, 37.85% were generally able to follow the lessons, and 42.53% could not follow the lessons due to poor communication activities with instructors^(12)^. Another study stated that 21% of the students followed all lessons^(27)^. Another study found that 50.9% of the students could not follow the lessons as they could not access a computer, and 32.6% of them could not reach the instructor for their questions^(5)^. We believe that strategies established in universities to increase the rate of course follow-up and opportunities for interaction with faculty members should increase SS.

In our research, it was determined that the satisfaction of students who think that DE is applicable for the health education and that theoretical lessons should be conducted with DE after the pandemic is positively affected. In the literature, it was stated that students believe that theoretical lessons in DE are efficient, while being inefficient for applied lessons, and they are not satisfied due to lack of question–answer opportunities and problems they face while entering the system^(28)^. Another study reported that, during the Covid-19, nursing students thought that DE would not be sufficient for both theoretical and applied lessons^(2)^. Integrating simulations used in health services into the DE process can increase satisfaction in applied lessons.

Our study found that a high level of digital literacy had a positive effect on educational satisfaction. Universities have adapted to the transition to DE programs during the pandemic. However, a comprehensive assessment and planning could not be conducted in terms of differences in students’ digital literacy levels^(11)^. In literature, it was stated that approximately two-thirds of students do not prefer DE using digital tools^(29)^, students’ digital literacy level is moderate^(3)^, and the lack of digital literacy in students is a major barrier for efficient digital learning^(30)^. We believe that after the pandemic, adding planned training to the university curricula for improving digital literacy levels will be beneficial. This, in turn, will increase students’ satisfaction with the educational model that is planned to be implemented after the pandemic and during the possible new pandemic processes.

We also found that the following did not affect educational satisfaction: students’ age, department, class, income status; having a Covid-19 test; family history of Covid-19; having a suitable environment for DE; tools of accessing DE; having difficulty in doing homework/presentation in the digital environment; demand to continue the applied lessons with DE after the pandemic; thinking that the pandemic limited access to the university library; thinking that DE would negatively affect the practice skills; and lifelong computer use,. Some studies determined that the sex variable did not affect SS^(5,7)^, and that the variables sex and having mobile internet have no effect on digital literacy skills^(3)^. Another study stated that male students have higher satisfaction with DE lessons compared to female students^(12)^; another one found that students’ age, computer usage frequency, department, total number of years they spend using a computer, and computer usage skills affect digital literacy skills^(3)^; and another one found that there is a significant relationship between the Covid-19 pandemic perception of students and the variables of age, their study fields, and sex; however, no significant relationship with the variable grade^(11)^ was observed. We believe that our study’s results can be used as a valuable input in planning of DE practices and formal education process during the pandemic.

## CONCLUSION

We conclude that students’ satisfaction with DE is moderate. In distance education, student satisfaction is reduced by variables such as living in the countryside; high perceived stress; low digital literacy; limited infrastructure opportunities, such as internet and computer access; and instructors’ not giving feedback. In this respect, improvements should be made to increase satisfaction with DE by focusing on factors that affect it. Steps need to be undertaken to completely resolve internet access problems for students living in areas with lack of technological infrastructure. Projects can be developed to provide computer support to students. Lessons on stress management and digital literacy can be added to the curriculum to reduce students’ stress and increase their digital literacy. Psychological support programs for university students to cope with stress and anxiety can be designed and sustained as part of the fight against the pandemic. To increase student’s satisfaction with distance education, platforms can be developed to increase the rate of students following courses on a regular basis and ensure effective interaction with instructors. This study can help guide future planning to improve the effectiveness of DE in universities and SS with DE.

### Strength and Limitation of Study

This study is one of the first to evaluate the effect of perceived stress and digital literacy on SS. However, the fact that the study was conducted in a single center limits its generalizability.
